# Periodic Variation of Mutation Rates in Bacterial Genomes Associated with Replication Timing

**DOI:** 10.1128/mBio.01371-18

**Published:** 2018-08-21

**Authors:** Marcus M. Dillon, Way Sung, Michael Lynch, Vaughn S. Cooper

**Affiliations:** a Department of Cell and Systems Biology, University of Toronto, Toronto, Ontario, Canada; b Graduate Program in Microbiology, University of New Hampshire, Durham, New Hampshire, USA; c Department of Bioinformatics and Genomics, University of North Carolina—Charlotte, Charlotte, North Carolina, USA; d Center for Mechanisms of Evolution, Arizona State University, Tempe, Arizona, USA; e Department of Microbiology and Molecular Genetics, University of Pittsburgh School of Medicine, Pittsburgh, Pennsylvania, USA; f Center for Evolutionary Biology and Medicine, University of Pittsburgh School of Medicine, Pittsburgh, Pennsylvania, USA; Massachusetts Institute of Technology

**Keywords:** Vibrio cholerae, Vibrio fischeri, genome organization, mutation rate, periodicity

## Abstract

The causes and consequences of spatiotemporal variation in mutation rates remain to be explored in nearly all organisms. Here we examine relationships between local mutation rates and replication timing in three bacterial species whose genomes have multiple chromosomes: Vibrio fischeri, Vibrio cholerae, and Burkholderia cenocepacia. Following five mutation accumulation experiments with these bacteria conducted in the near absence of natural selection, the genomes of clones from each lineage were sequenced and analyzed to identify variation in mutation rates and spectra. In lineages lacking mismatch repair, base substitution mutation rates vary in a mirrored wave-like pattern on opposing replichores of the large chromosomes of V. fischeri and V. cholerae, where concurrently replicated regions experience similar base substitution mutation rates. The base substitution mutation rates on the small chromosome are less variable in both species but occur at similar rates to those in the concurrently replicated regions of the large chromosome. Neither nucleotide composition nor frequency of nucleotide motifs differed among regions experiencing high and low base substitution rates, which along with the inferred ~800-kb wave period suggests that the source of the periodicity is not sequence specific but rather a systematic process related to the cell cycle. These results support the notion that base substitution mutation rates are likely to vary systematically across many bacterial genomes, which exposes certain genes to elevated deleterious mutational load.

## IMPORTANCE

That mutation rates vary within bacterial genomes is well known, but the detailed study of these biases has been made possible only recently with contemporary sequencing methods. We applied these methods to understand how bacterial genomes with multiple chromosomes, like those of *Vibrio* and *Burkholderia*, might experience heterogeneous mutation rates because of their unusual replication and the greater genetic diversity found on smaller chromosomes. This study captured thousands of mutations and revealed wave-like rate variation that is synchronized with replication timing and not explained by sequence context. The scale of this rate variation over hundreds of kilobases of DNA strongly suggests that a temporally regulated cellular process may generate wave-like variation in mutation risk. These findings add to our understanding of how mutation risk is distributed across bacterial and likely also eukaryotic genomes, owing to their highly conserved replication and repair machinery.

## INTRODUCTION

Mutation rates may vary within genomes for a variety of reasons, from straightforward causes like repetitive sequences causing polymerase slippage or the deamination and errant repair of methylated bases to more complex causes like transcription-translation conflicts (1, 2). These processes tend to produce mutation rate heterogeneity over intervals less than 1 kb. What is underappreciated is the potential for mutation rates to vary over longer ranges that may exceed 100 kb and affect hundreds of genes. The prevalence and causes of long-range variation are unclear but have been attributed to effects of error-prone polymerases (3), error-prone repair pathways (4), and inconsistent nucleotide pools (5). If this long-range variation is common and systematic, the affected genes would be subject to greater mutational load, and this process could select for gene reordering to avoid mutation risk.

On the other hand, replication timing, or the relative distance from the origin of replication, is one of the most conserved properties of orthologous genes (6). Selection to maintain gene order has been attributed mostly to gene expression, where intragenic variation in the binding of nucleotide-associated proteins (NAPs) and compaction of the nucleoid induce selection on gene order and location for optimal expression (6–9). Consequently, genes may face conflicts between the demand for optimal expression and their mutation risk, which has broad implications for genome evolution and genetic diseases. A series of comparative studies in multicellular eukaryotes (10–13), unicellular eukaryotes (12, 14), archaea (15), and bacteria (16, 17) have shown that synonymous substitution rates—a product of all population genetic forces, including mutation, genetic drift, and selection—vary across the genome and generally increase in late-replicating regions. This correlation could result from higher base substitution mutation (bpsm) rates or weaker purifying selection in late-replicating regions (1, 16, 18). A powerful approach to disentangle these processes is the mutation accumulation (MA) experiment analyzed by whole-genome sequencing (WGS), in which many replicate lineages are passaged through hundreds of single-cell bottlenecks in the near absence of natural selection and all mutations are identified. Our aim was to directly test whether *de novo* mutation rates vary among genome regions and specifically whether such long-range systematic mutation rate variation operates in bacteria.

This study builds upon several prior MA-WGS studies in diverse bacterial species. Above all, mutation rates in bacteria are remarkably low, even dropping below 10^−3^/genome/generation (1, 19). Such low rates mean that MA experiments using wild-type strains with intact mismatch repair (MMR) fail to capture enough mutations to detect long-range mutation rate variation (19–21). MA studies with MMR-deficient organisms generate much larger collections of mutations but have shown no simple, linear correlation between bpsm rates and replication timing (19, 22–25). Thus, the more rapid evolution of late-replicated genes likely results from weaker purifying selection, not increased mutation rates. More intriguingly, MA studies of MMR-deficient bacteria, including Escherichia coli, Pseudomonas fluorescens, Pseudomonas aeruginosa, and Bacillus subtilis, have revealed significant nonlinear or periodic variation in mutation rates among genome regions (19, 22–25).

We chose to study three bacterial species with genomes containing multiple circular chromosomes: Vibrio cholerae, Vibrio fischeri, and Burkholderia cenocepacia (19, 21). This is an underappreciated but not uncommon bacterial genome architecture (16, 26–28) and enables effects of chromosome location and replication timing to be distinguished. Setting aside the distinction between chromosomes and megaplasmids (29), the Vibrio cholerae and Vibrio fischeri genomes are composed of two chromosomes, while the Burkholderia cenocepacia genome is composed of three. In each species, the first chromosome (chr1) is the largest, harbors the most essential genes, and is expressed at the highest levels (16, 30). Secondary chromosomes (chr2 and chr3) also initiate replication from a single origin and are replicated bidirectionally on two replichores (28, 31, 32). While they are replicated at the same rate as the first chromosome, their origins of replication (*oriCII*) have distinct initiation requirements from those of chr1 origins (*oriCI*) (26, 33). Importantly, chr2 (or chr3) replication is delayed relative to chr1 to ensure that replication of all chromosomes terminates synchronously (28, 32, 34). Consequently, the genome region near the origin of chr1 is always replicated prior to secondary chromosomes, while late-replicated regions of chr1 are replicated concurrently with chr2.

This replication timing program in bacteria with multiple circular chromosomes enabled a test of whether secondary chromosomes experience similar mutation rates and regional variation to concurrently late-replicated regions of primary chromosomes. Here we report detailed analyses of the genome-wide distribution of spontaneous bpsms generated by MA-WGS experiments with MMR-deficient strains of V. fischeri (4,313 bpsms) and V. cholerae (1,022 bpsms), as well as spontaneous bpsms generated by MA-WGS experiments with MMR-proficient strains of V. fischeri (219 bpsms), V. cholerae (138 bpsms), and B. cenocepacia (245 bpsms) (19, 21). We define the patterns of fluctuations in mutation rates within each genome and assess whether this variance affects coordinately replicated regions within and among chromosomes. In the MMR-deficient lines, we find evidence of systematic variation in mutation rate that implies that the causative factors act not just spatially but also temporally with the cell cycle, a phenomenon that could apply to a broad range of organisms.

## RESULTS

Two MMR-deficient (mutator, MMR^−^) and three MMR-proficient (wild-type) MA-WGS experiments were founded with five different ancestral strains: (i) the V. fischeri ES114 Δ*mutS* mutant, (ii) the V. cholerae 2740-80 Δ*mutS* mutant, (iii) the V. fischeri ES114 wild type, (iv) the V. cholerae 2740-80 wild type, and (v) the B. cenocepacia HI2424 wild type. Forty-eight independent MA lineages were propagated for 43 days in the two mutator experiments, and 75 MA lineages were propagated for 217 days in the three wild-type experiments. In total, successful WGS was completed on evolved clones of 19 V. fischeri and 22 V. cholerae MMR^−^ lineages and 48 V. fischeri, 49 V. cholerae, and 47 B. cenocepacia wild-type lineages. Despite the fact that the mutator experiments were shorter and involved fewer lineages, the vast majority of bpsms were generated in the V. fischeri and V. cholerae MMR^−^ lineages, as their bpsm rates are 317-fold and 85-fold greater than those of their wild-type counterparts, respectively. Consequently, effects of genomic position on bpsm rates can be studied in much greater detail in the mutator lineages, where adequate numbers of bpsms are distributed across the genome at intervals as short as 10 kb (Table 1), the approximate length of bacterial microdomains (7).

**TABLE 1:** Number of bpsms in each mutation accumulation experiment and the associated average number of bpsms in intervals of various sizes

MA line	No. of bpsms	No. of bpsms at interval of:											
		500 kb		250 kb		100 kb		50 kb		25 kb		10 kb	
		Avg	SEM	Avg	SEM	Avg	SEM	Avg	SEM	Avg	SEM	Avg	SEM
MMR^−^													
V. fischeri	4,313	499.00	35.82	253.50	13.77	101.05	3.12	50.53	1.26	25.26	0.51	10.08	0.18
V. cholerae	1,022	141.50	21.14	65.33	6.23	25.47	1.53	12.51	0.59	6.22	0.25	2.50	0.09
Wild type													
V. fischeri	219	22.25	3.28	12.25	1.39	4.95	0.40	2.48	0.20	1.24	0.09	0.50	0.03
V. cholerae	138	18.00	3.09	8.75	0.95	3.42	0.30	1.72	0.14	0.83	0.07	0.34	0.03
B. cenocepacia	245	15.90	1.43	7.58	0.57	3.27	0.22	1.62	0.11	0.81	0.05	0.32	0.02

In comparing the overall bpsm rates between chromosomes in the mutator lineages, we observed that the bpsm rates on chr1 and chr2 of V. fischeri were not statistically distinguishable (χ^2^ = 0.11, df = 1, *P* = 0.741), while the bpsm rate on chr1 of V. cholerae was slightly higher than the rate on chr2 (χ^2^ = 4.54, df = 1, *P* = 0.0331) (19). However, even in V. cholerae, the variation in bpsm rates was minimal between chromosomes, and our data suggested that considerably greater variation may exist within chromosomes (19). To determine the effects of genomic position on bpsm rates on a finer scale, we analyzed bpsm rates among intervals of various sizes (10 to 500 kb) extending bidirectionally from the *oriCI* as the replication forks proceed during replication. Rates on chr2 were analyzed using the same intervals as chr1 but according to the inferred replication timing of *oriCI* (see Fig. S1A in the supplemental material). This enables direct comparisons between concurrently replicated intervals on both chromosomes. To illustrate how this analysis works, we plotted the patterns of bpsm rates from a recent E. coli mutator MA experiment in which mutation rates were demonstrated to vary in a wave-like pattern that is mirrored on the two replichores of its singular circular chromosome (20, 22) (Fig. S1B). If replication timing is responsible for this pattern, a hypothetical secondary chromosome in E. coli would be expected to mirror concurrently replicated (late-replicating) regions on the primary chromosome (Fig. S1B).

10.1128/mBio.01371-18.2 FIG S1  Design of the interval analysis used in this study to enable direct comparisons of base substitution mutation (bpsm) rates of concurrently replicated regions on chromosome 1 (chr1) and chromosome 2 (chr2). (A) For all multichromosome species analyzed in this study, secondary chromosomes are split at their origin of replication (*oriCII*) and mapped directly to concurrently replicated intervals in late-replicating regions of chr1. All intervals on both chromosomes are thus relative to the initiation of replication of *oriCI*, and the boundaries of the intervals are consistent with their replication timing. (B) Patterns of bpsm rates on the single chromosome of Escherichia coli MG1655 *rph*^+^ Δ*mutL*, derived from reference 20, show a wave-like mirrored pattern of bpsm rates on the two opposing replichores. If replication timing governs this pattern, a hypothetical secondary chromosome would be expected to mirror patterns of bpsm rates of late-replicated regions on the primary chromosome. Download FIG S1, PDF file, 0.3 MB. Copyright © 2018 Dillon et al. 2018 Dillon et al. This content is distributed under the terms of the Creative Commons Attribution 4.0 International license.

### Base substitution mutation rates are wave-like on chr1 in mutator lines.

**FIG 1:**
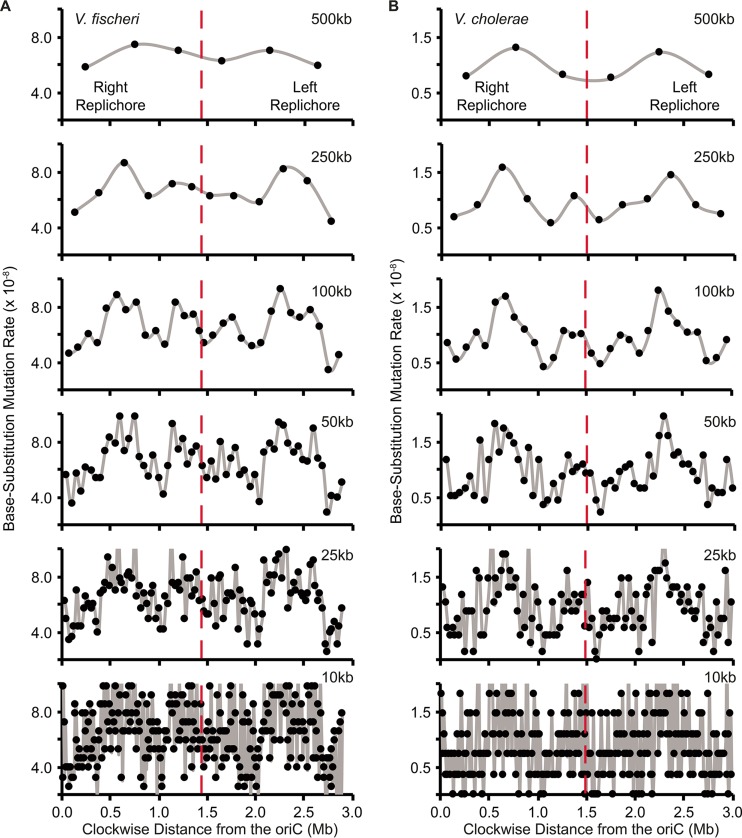
Patterns of base substitution mutation (bpsm) rates at various size intervals extending clockwise from the origin of replication (*oriC*) in MMR-deficient mutation accumulation lineages of V. fischeri (A) and V. cholerae (B) on chromosome 1. bpsm rates are calculated as the number of mutations observed within each interval divided by the product of the total number of sites analyzed within that interval across all lines and the number of generations of mutation accumulation. The two intervals that meet at the terminus of replication (dotted red line) on each replichore are shorter than the interval length for that analysis, because the size of chromosome 1 is never exactly divisible by the interval length.

**FIG 2:**
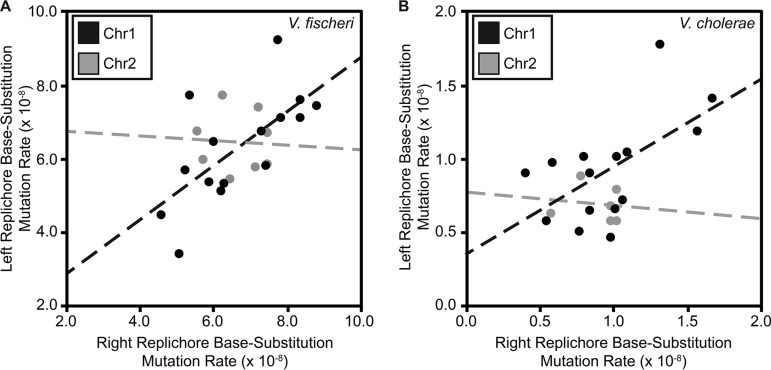
Relationship between base substitution mutation (bpsm) rates in 100-kb intervals on the right replichore with concurrently replicated 100-kb intervals on the left replichore in MMR-deficient Vibrio fischeri (A) and Vibrio cholerae (B). Both linear regressions are significant on chr1 (V. fischeri, *F* = 10.98, df = 13, *P* = 0.0060, *r*^2^ = 0.46; V. cholerae, *F* = 6.76, df = 13, *P* = 0.0221, *r*^2^ = 0.34) but not on chr2 (V. fischeri, *F* = 0.02, df = 6, *P* = 0.8910, *r*^2^ = 0.03 × 10^−1^; V. cholerae, *F* = 0.06, df = 4, *P* = 0.8140, *r*^2^ = 0.02).

Mutation rates were not uniformly distributed across 10- to 500-kb intervals on chr1 in either the V. fischeri or V. cholerae MMR^−^ MA experiments (see Data Set S1 in the supplemental material), but we could not reject the null hypothesis of uniform rates on chr2, which has lower variance in bpsm rates. Variation in bpsm rates on chr1 in both V. fischeri and V. cholerae MMR^−^ experiments follows a wave-like pattern that is mirrored on both replichores bidirectionally from the origin of replication (Fig. 1A and B). This mirrored pattern is evident at multiple interval sizes and is consistent with what has been reported on the single chromosome of E. coli (22), although the lengths of the wave periods observed here are shorter (Fig. S1B). The waveform of bpsm rates is low near *oriCI*, increases to its peak approximately 600 kb from the *oriCI* on both replichores, and declines into another valley before rising and falling again in the approach to the replication terminus. Two distinct waves can be seen on each replichore of chr1 (Fig. 1A and B) but are less evident on chr2 (see Fig. S2 in the supplemental material). We focused our most detailed analyses of patterns of bpsm rate variation at the 100-kb interval because it maximizes the number of bpsms/interval while retaining two apparently mirrored bpsm rate waves on each replichore. Over 100-kb intervals, we see a significantly positive correlation between bpsm rates of concurrently replicated regions on the left and right replichores of chr1 in both V. fischeri and V. cholerae (Fig. 2A and B). This relationship is also significant at most other interval lengths (Data Set S1), but we find no such relationship when comparing 100-kb intervals on the left and right replichores of chr2 as a consequence of its lower variance in bpsm rate.

10.1128/mBio.01371-18.3 FIG S2  Patterns of base substitution mutation (bpsm) rates at various size intervals extending clockwise from the origin of replication (*oriCII*) in MMR-deficient mutation accumulation lineages of Vibrio fischeri (A) and Vibrio cholerae (B) on chromosome 2. All interval breakpoints are plotted relative to the initiation of replication of *oriCI* so that the boundaries of the intervals are at identical locations. Download FIG S2, PDF file, 0.4 MB. Copyright © 2018 Dillon et al. 2018 Dillon et al. This content is distributed under the terms of the Creative Commons Attribution 4.0 International license.

10.1128/mBio.01371-18.8 DATA SET S1  Summary of all base substitution mutations identified in each of the five mutation accumulation experiments carried out for this study, χ^2^ statistics of tests for uniform mutation rates, linear regression statistics for correlations between replichores and chromosomes, and residual fit of mutation rates to different chromosome intervals. Download DATA SET S1, XLSX file, 0.5 MB. Copyright © 2018 Dillon et al. 2018 Dillon et al. This content is distributed under the terms of the Creative Commons Attribution 4.0 International license.

### Concurrently replicated regions between chromosomes exhibit similar mutation rates.

**FIG 3:**
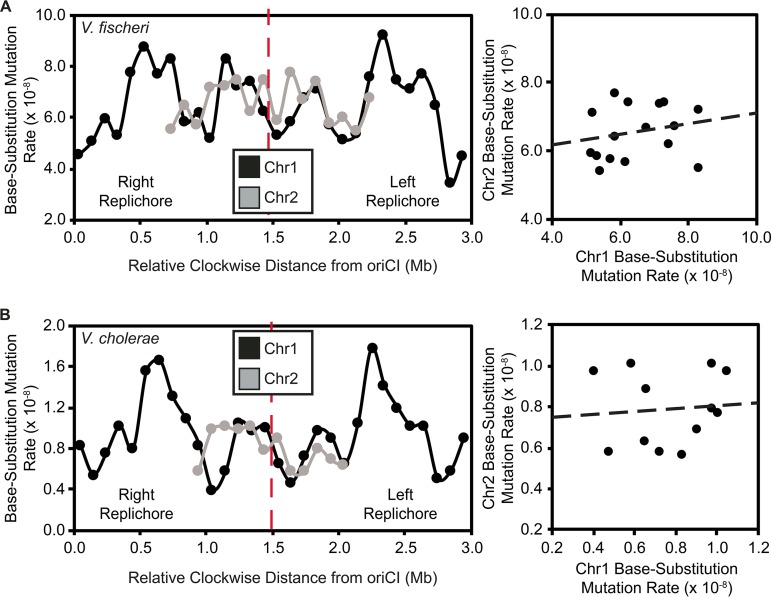
Patterns of base substitution mutation (bpsm) rates in 100-kb intervals extending clockwise from the origin of replication (*oriCI*) on chromosome 1 (chr1) and patterns of bpsm rates of concurrently replicated 100-kb intervals on chromosome 2 (chr2) for MMR-deficient Vibrio fischeri (A) and Vibrio cholerae (B). Patterns of bpsm rates on chr2 appear to map to those of concurrently replicated regions on chr1 in both species, but the linear regressions between concurrently replicated intervals are not significant on chr1 and chr2 in either V. fischeri or V. cholerae (V. fischeri, *F* = 0.62, df = 14, *P* = 0.4442, *r*^2^ = 0.04; V. cholerae, *F* = 0.07, df = 10, *P* = 0.7941, *r*^2^ = 0.01).

Given the observed relationship between bpsm rates of concurrently replicated regions on chr1, we might also expect late-replicated regions of chr1 to experience similar bpsm rates to chr2 because of their concurrent replication. To study this relationship, we mapped the patterns of bpsm rates in 100-kb intervals on chr2 to those of late-replicated 100-kb intervals on chr1 for both the V. fischeri and V. cholerae MMR^−^ experiments (Fig. S1). Fluctuations in bpsm rates on chr2 resemble those of late-replicated regions on chr1 in both species (Fig. 3A and B), but linear correlations in bpsm rates between chr1 and chr2 were not significant (Data Set S1). However, this lack of significant relationship may be a reflection of late-replicated regions generally experiencing lower variance in bpsm rates than chr1 as a whole, and given the strong resemblance in bpsm rate fluctuations between chr2 and concurrently late-replicated regions of chr1, we attempted to falsify this match by correlating chr2 bpsm rates by correlating chr2 bpsm rates with all possible interval combinations on the right and left replichores of chr1. For the V. fischeri MMR^−^ experiment, the lowest sum of the residuals (14.01 × 10^−8^) occurs when the chr2 intervals were mapped to the concurrently late-replicated intervals on chr1 (Fig. 3A; Data Set S1). The same pattern was found for the V. cholerae MMR^−^ experiment (Fig. 3B; Data Set S1). Thus, despite no significant linear correlation in mutation rate periodicity between chr1 and chr2, the spatial variation in bpsm rates on chr2 most closely resembles the rates of concurrently replicated regions on chr1 in both V. cholerae and V. fischeri. Interestingly, the delayed replication and small size of chr2 allow it to narrowly avoid the peak bpsm rates on the right and left replichores of chr1 in both the V. fischeri and V. cholerae MMR^−^ experiments (Fig. 3A and B). Thus, genes on chr2 may be subjected to a less deleterious load than many of the genes on chr1, particularly in V. cholerae.

### Wavelet transformations capture periodicities in base substitution mutation rates.

**FIG 4:**
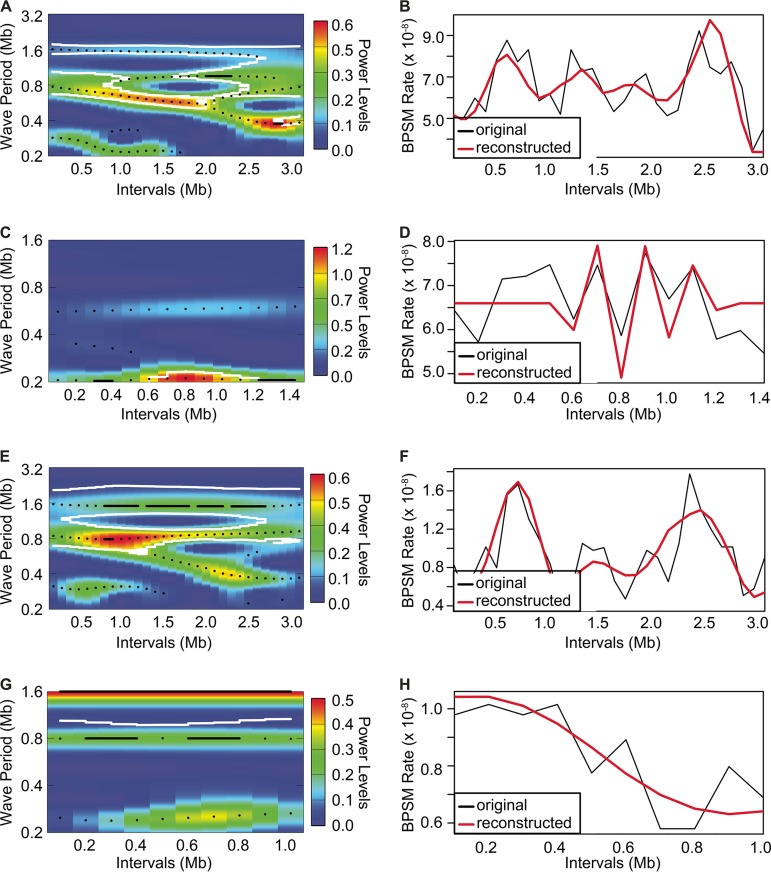
Wavelet power spectrum and resultant reconstruction of the patterns of base substitution mutation (bpsm) rates in 100-kb intervals extending clockwise from the *oriCI* region of chromosome 1 (A and B, V. fischeri; E and F, V. cholerae) and the *oriCII* region of chromosome 2 (C and D, V. fischeri; G and H, V. cholerae) using the MMR-deficient mutation accumulation lineages. Wavelet power analyses follow an interval color key (A, C, E, and G), where colors code for the power values at each interval in the genome for all possible wave periods, from dark blue (low power) to dark red (high power). White contour lines denote a significance cutoff of 0.1. Reconstructed series were generated using only the wave periods whose average power was significant over the entire interval (B, D, F, and H).

Recognizing that regional or cyclic variation in mutation rates may not be captured by linear models, we used wavelet transformations to characterize periodicities in the mirrored wave-like patterns in bpsm rates observed in this study. The bpsm rates on each chromosome from the V. fischeri and V. cholerae MMR^−^ experiments were transformed using the Morlet wavelet (35), which can reveal time-associated changes in the frequency of bpsms and has been successfully used in ecologic time series analyses (36). This method was used to identify significant wave periods in bpsm rates on chr1 and chr2 and any variation in period length or amplitude across the chromosome. Significant wave periods of approximately 1.6 and 0.8 Mb extend clockwise from *oriCI* in the V. fischeri MMR^−^ lineages (Fig. 4A). The single long-period wave of 1.6 Mb is well supported across each replichore, while the shorter ~0.8-Mb period wave is significant across most of chr1, but its inferred length varies between 0.6 and 1.0 Mb. Thus, there are two synchronous periods per replichore, or four periods in total around the chromosome, which are also clearly evident in Fig. 1. These two wave periods of approximately 1.6 and 0.8 Mb were also observed in the bpsm rate data found on chr1 in the V. cholerae MMR^−^ lineages (Fig. 4E).

Using only these wave models, we successfully reproduced the apparent periodicity of the 100-kb data in both the V. fischeri and V. cholerae MMR^−^ lineages (Fig. 4B and F). Next, using the cross-wavelet transformation method to identify shared periodicities between replichores (35), we found that the wave model derived from one replichore predicts the behavior of the other (see Fig. S3A and B in the supplemental material). It is also noteworthy that the statistically synchronous waves become smaller near the replication terminus, particularly in the V. fischeri mutator lineages (Fig. S3A and B), which is also apparent in the raw data presented in Fig. 1. Perhaps because of this lower variation in late-replicated regions, these modeling efforts were not successful on chr2 for either the V. fischeri or V. cholerae MMR^−^ experiment (Fig. 4C and D and G and H).

10.1128/mBio.01371-18.4 FIG S3  Cross-wavelet power spectrum plots comparing the patterns of base substitution mutation (bpsm) rates in 100-kb intervals extending clockwise from the *oriCI* region to those extending counterclockwise from the *oriCI* region in MMR-deficient mutation accumulation lineages of Vibrio fischeri (A) and Vibrio cholerae (B). Plots were generated using the WaveletComp package for Computational Wavelet Analysis in R using an interval color key, 100 simulations, and significant synchronicity cutoffs of *P* < 0.1 for contour (white lines) and *P* < 0.05 for arrows. Colors represent the cross-wavelet power values at each interval in the genome for all possible wave periods, from dark blue (low power) to dark red (high power). Download FIG S3, PDF file, 0.3 MB. Copyright © 2018 Dillon et al. 2018 Dillon et al. This content is distributed under the terms of the Creative Commons Attribution 4.0 International license.

### Replication-associated periodicity results from specific forms of base substitution mutations.

Nucleotide content varies across chromosomes and could conceivably underlie variation in bpsm rates among 100-kb intervals. To address this possibility, we focused on A⋅T > G⋅C and G⋅C > A⋅T transitions in the V. fischeri and V. cholerae MMR^−^ studies, as these two forms of bpsm represent 97.93% and 98.34% of all observed bpsms, respectively (19). Nucleotide composition did not vary significantly among 100-kb intervals on chr1 or chr2. However, the spectra of bpsms corrected for nucleotide content varied significantly among intervals on chr1 in both the V. fischeri and V. cholerae MMR^−^ MA experiments (χ^2^ test; A⋅T > G⋅C, V. fischeri MMR^−^, χ^2^ = 62.26, df = 29, *P* = 0.0003; V. cholerae MMR^−^, χ^2^ = 49.04, df = 29, *P* = 0.0110; G⋅C > A⋅T, V. fischeri MMR^−^, χ^2^ = 120.69, df = 29, *P* < 0.0001; V. cholerae MMR^−^, χ^2^ = 111.19, df = 29, *P* < 0.0001). On chr2, only G⋅C > A⋅T substitutions in the V. fischeri MMR^−^ experiment varied among intervals (χ^2^ = 26.81, df = 15, *P* = 0.0300). Interestingly, G⋅C > A⋅T mutation rates exhibit the greatest variation among chr1 intervals in both the V. fischeri and V. cholerae MMR^−^ studies, and the positive correlations in bpsm rates on opposing replichores are driven largely by G⋅C > A⋅T, not A⋅T > G⋅C, bpsms (see Fig. S4 in the supplemental material). The periodicity in bpsm rates in the V. fischeri and V. cholerae MMR^−^ lines is therefore not caused by differences in nucleotide content but is predominantly caused by G⋅C > A⋅T transitions.

10.1128/mBio.01371-18.5 FIG S4  Relationship between base substitution mutation (bpsm) rates in 100-kb intervals on the right replichore with concurrently replicated 100-kb intervals on the left replichore for A⋅T > G⋅C (A) and G⋅C > A⋅T (B) bpsms in MMR-deficient Vibrio fischeri and A⋅T > G⋅C (C) and G⋅C > A⋅T (D) bpsms in MMR-deficient Vibrio cholerae. Only the relationship between G⋅C > A⋅T bpsm rates of concurrently replicated regions on chr1 is significantly positive (V. fischeri, A⋅T > G⋅C, chr1, *F* = 1.77, df = 13, *P* = 0.2067, *r*^2^ = 0.12, and chr2, *F* = 3.26, df = 6, *P* = 0.1209, *r*^2^ = 0.35; G⋅C > A⋅T, chr1, *F* = 13.32, df = 13, *P* = 0.0029, *r*^2^ = 0.51, and chr2, *F* = 0.17, df = 6, *P* = 0.6947, *r*^2^ = 0.03; V. cholerae, A⋅T > G⋅C, chr1, *F* = 0.24, df = 13, *P* = 0.6313, *r*^2^ = 0.02, and chr2, *F* = 1.74, df = 4, *P* = 0.2574, *r*^2^ = 0.30; G⋅C > A⋅T, chr1, *F* = 28.99, df = 13, *P* = 0.0001, *r*^2^ = 0.6904, and chr2, *F* = 0.15, df = 4, *P* = 0.7209, *r*^2^ = 0.04). Download FIG S4, PDF file, 0.4 MB. Copyright © 2018 Dillon et al. 2018 Dillon et al. This content is distributed under the terms of the Creative Commons Attribution 4.0 International license.

The immediate 5′ and 3′ nucleotide context of the mutated base can also influence rates and could conceivably lead to periodicity if trimers vary among intervals. Indeed, genome-wide bpsm rates in both the V. fischeri and V. cholerae MMR^−^ studies vary more than 50-fold, depending on the 5′ and 3′ bases flanking the site of the bpsm (see Fig. S5 in the supplemental material). This phenomenon has been found in several bacterial genomes and was found to be driven by sites neighboring G⋅C base pairs or dimers, including alternating pyrimidine-purine and purine-pyrimidine nucleotides having significantly elevated mutation rates (24). However, the product of trimer abundance and specific mutation rates cannot explain the distribution of bpsms measured here on chr1 in either V. cholerae or V. fischeri (Fig. S5).

10.1128/mBio.01371-18.6 FIG S5  Effects of nucleotide context (trimer content) on bpsm rates. (A) Heat map of the context-dependent base substitution mutation (bpsm) rates for the 64 possible trimer combinations based on their lagging strand orientation in MMR-deficient mutation accumulation lineages of Vibrio fischeri (A) and Vibrio cholerae (B). (B) Patterns of base substitution mutation (bpsm) rates in 100-kb intervals extending clockwise from the origin of replication (*oriCI*) in MMR-deficient mutation accumulation lineages of Vibrio fischeri (A) and Vibrio cholerae (B). Observed patterns of bpsm rates (gray lines) on chromosome 1 (chr1) and chromosome 2 (chr2) are compared to the expected patterns of bpsm rates (blue lines) based on the trimer content of the interval. bpsm rates differ significantly from expectations based on trimer content: χ^2^ test; V. fischeri MMR^−^, chr1, χ^2^ = 137.24, df = 29, *P* < 0.0001, and chr2, χ^2^ = 20.04, df = 15, *P* = 0.1703; V. cholerae MMR^−^, chr1, χ^2^ = 107.55, df = 29, *P* < 0.0001, and chr2, χ^2^ = 14.87, df = 1, *P* = 0.1887). Download FIG S5, PDF file, 0.6 MB. Copyright © 2018 Dillon et al. 2018 Dillon et al. This content is distributed under the terms of the Creative Commons Attribution 4.0 International license.

### Low base substitution mutation rates in wild-type lineages reveal modest regional variation.

**FIG 5:**
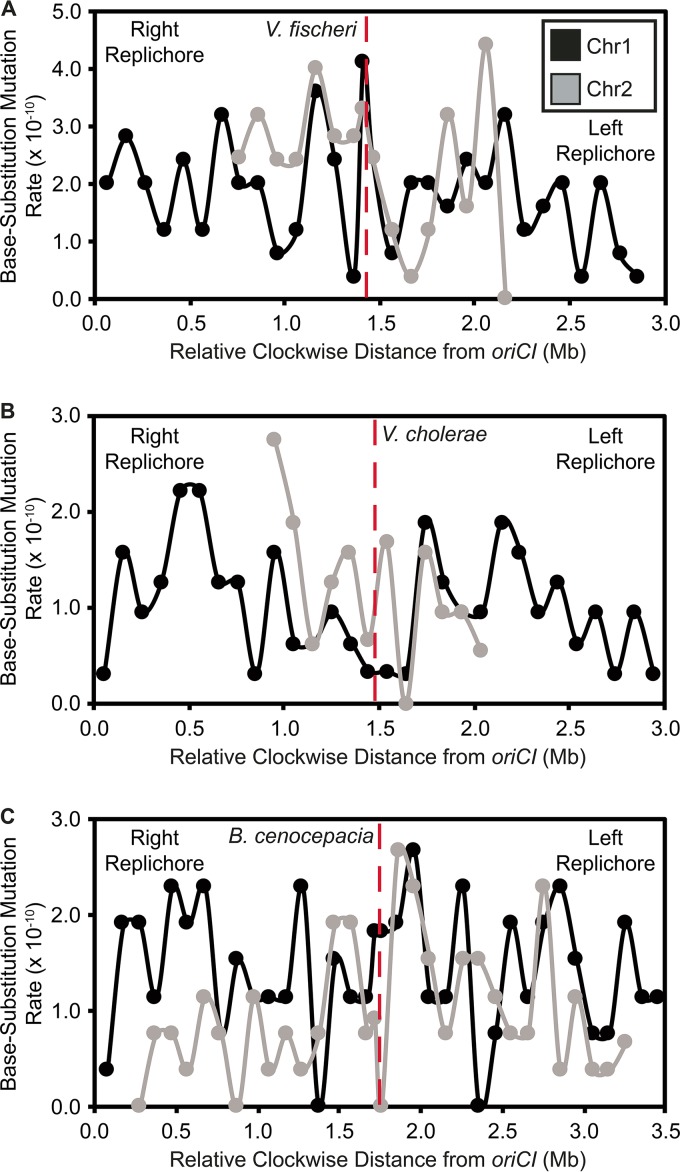
Patterns of base substitution mutation (bpsm) rates in 100-kb intervals extending clockwise from the origin of replication (*oriC*) on chromosome 1 (chr1) and concurrently replicated intervals of chromosome 2 (chr2) for WT (MMR^+^) Vibrio fischeri (A), Vibrio cholerae (B), and Burkholderia cenocepacia (C). B. cenocepacia also has a third chromosome, which is not shown. These visual patterns are not statistically significant, perhaps owing to low sample size: (linear regression; V. fischeri wild type, *F* = 0.16, df = 14, *P* = 0.7001, *r*^2^ = 0.01; V. cholerae wild type, *F* = 2.72, df = 10, *P* = 0.1300, *r*^2^ = 0.21; B. cenocepia wild type, *F* = 0.32, df = 30, *P* = 0.5760, *r*^2^ = 0.01).

Despite conducting longer MA experiments (217 days versus 43 days) and sequencing more lineages (48 versus 22) derived from wild-type, MMR^+^ ancestors of V. fischeri, V. cholerae, and B. cenocepacia, considerably fewer bpsms accumulated in these lines than in MMR^−^ lines. Consequently, we cannot reject the null hypothesis that bpsms are uniformly distributed across chr1, chr2, and chr3 (for B. cenocepacia) in the V. fischeri, V. cholerae, or B. cenocepacia wild-type MA experiments (Data Set S1). Furthermore, coordinately replicated regions of chr1 and chr2 also did not exhibit correlated mutation rates, likely because of low sample sizes (Fig. 5A and B and C). Only means of 4.65 (standard error of the mean [SEM], 0.38), 3.29 (SEM, 0.28), and 3.08 (SEM, 0.22) bpsms per 100-kb interval were detected for the V. fischeri, V. cholerae, and B. cenocepacia wild-type MA lineages, respectively. Using effect size estimates derived from the significant patterns in MMR^−^ lines (see Text S1 in the supplemental material), we estimate that the 132 mutations found on chr1 in the V. fischeri wild-type experiment would reveal a significantly nonuniform distribution of bpsms in only 19.46% of cases. The same analysis applied to the V. cholerae wild-type experiment predicts that significant regional variation in bpsms would be identified only 43.95% of the time. Furthermore, applying effects from the V. cholerae MMR^−^ experiment to the B. cenocepacia wild-type experiment suggests that significant regional variation would be seen on chr1 in 55.16% of cases. Greater experimental replication may be needed to capture more mutations in wild-type genomes to determine whether the periodicity in mutation rates seen in mutator lines also occurs in wild-type genomes, but we did observe that the patterns of bpsm rate variation in the V. cholerae wild-type experiment, where the effect size was largest, correlate with that of the corresponding mutator experiment, which implies a common underlying process for variation in mutator and wild-type bpsm rates (linear regression, 100-kb intervals; V. cholerae wild type-V. cholerae MMR^−^, *F* = 5.07, df = 38, *P* = 0.0303, *r*^2^ = 0.12).

10.1128/mBio.01371-18.1 TEXT S1  Supplemental methods. Download TEXT S1, DOCX file, 0.1 MB. Copyright © 2018 Dillon et al. 2018 Dillon et al. This content is distributed under the terms of the Creative Commons Attribution 4.0 International license.

## DISCUSSION

Variation in mutation rates among genome regions can have important implications for genome evolution and diseases, including most cancers (6–8, 37–40). One of the most conserved properties of genome organization is the relative distance of genes from the origin of replication (6, 41), which is expected to result in the long-term conservation of traits like expression and mutation rates for genes harbored in divergent genomes. Consequently, molecular modifications that change genome-wide patterns of replication timing, expression, and mutation rates could increase the probability of acquiring defective alleles in typically conserved regions, leading to disease. Indeed, alteration of the replication timing program can be an early step in carcinogenesis and a number of other somatic disease states (37). However, given the remarkable diversity in genome architecture across the tree of life, we still have much to learn about the nature of regional patterns of variation in bpsm rates and the genomic features and molecular processes that govern them.

Periodic variation in bpsm rates that is mirrored on the two replichores of bacterial chromosomes has been observed in genomes of some single-chromosome bacteria that are MMR deficient (22, 25), yet not all species appear to experience this periodicity (23), and the underlying causes of periodic variation in bacterial bpsm rates are unknown. Here we demonstrate that MMR-deficient bacterial genomes with multiple chromosomes display mirrored, wave-like patterns of bpsm rates on chr1 (Fig. 1A and B), and although we cannot reject the null hypothesis that bpsm rates are uniform on chr2, the patterns of bpsm rates on chr2 best match those of concurrently replicating regions on chr1 (Fig. 3A and B). Furthermore, much of the genome-wide variation in bpsm rates that we observe appears to be generated by G⋅C > A⋅T transitions in both the V. fischeri and V. cholerae MMR^−^ studies. Three MA experiments with MMR-proficient genomes hint at regional variation in bpsm rates, but these studies were insufficiently powered to reject the null hypothesis of uniformity. Nonetheless, shared periodicities in mutation rates between replichores and coarse similarities across chromosome regions that are coordinately replicated suggests strongly that mutation rates are affected by one or more common, global processes. Such a process influences replication fidelity throughout the genome at different active replication forks and causes bpsm rates to occur at a minimum level near the replication origin, rise to roughly 2 to 4 times these rates, and then decline and repeat this cycle before replication termination. If physically separate genome regions share common mutation rates because of their shared replication timing, their genetic content may also be subject to common evolutionary forces.

This study cannot directly test the potential causes of mutation rate variation, but the bpsm patterns are more consistent with certain causes. First, nucleotide context can generate heterogeneous bpsm rates because certain nucleotides or nucleotide contexts are more prone to incur bpsms than others (20, 23, 24, 42, 43), and there is reason to believe that concurrently replicated regions on opposing replichores contain symmetrical gene content (41). Although we find that bpsm rates in both the V. fischeri and V. cholerae MMR^−^ studies vary more than 50-fold depending on the bases flanking the site of the bpsm (Fig. S5), this variation cannot explain the overall rate periodicity.

The replication machinery itself may also generate heterogeneous bpsm rates because of biased usage of error-prone polymerases (3) or repair pathways (4) in certain genome regions. Both mechanisms have been invoked to explain why substitution rates scale positively with replication timing (4, 10–17), but the majority of these studies were performed in eukaryotes, and it is difficult to imagine how they might create the mirrored wave-like patterns of bpsm rates observed in bacterial chromosomes across 100-kb intervals. Indeed, a series of MA studies in E. coli have shown that error-prone polymerases have minimal effects on mutation rates in the absence of DNA damage or stress (44).

Other genomic features that vary systematically with replication timing like binding of nucleoid-associated proteins (NAPs), transcription levels, and compaction of the bacterial nucleoid are also candidates for explaining our observed patterns of bpsm rates (6–9, 45). Sigma factors, DNA gyrase, and a number of NAPs have mirrored patterns of activity on the right and left replichores of the single chromosome in E. coli (6), possibly resulting from their concurrent replication. The resultant negative DNA superhelicity does correlate positively with the mirrored wave-like patterns of bpsm rates on opposing replichores of E. coli (22), and patterns of extant sequence variation are significantly impacted by NAPs that bind the DNA at different growth phases (9). However, effects of NAPs on sequence variation among published genomes are relatively weak and unlikely to produce the 2- to 4-fold changes in bpsm rates observed across the long interval lengths used in this study (9). While transcription levels may also impact bpsm rates through gene expression and replication-transcription conflicts (46), oscillations in expression patterns and gene density are not consistent with concurrently replicated regions experiencing similar expression levels (47), and expression has not been significantly correlated with the patterns of bpsm rates in E. coli and other species (1, 22).

The G⋅C > A⋅T and G⋅C > T⋅A bpsms that drive much of the observed periodicity are consistent with damage induced by reactive oxygen species (ROS) (Fig. S4). It is conceivable that the plate growth conditions in these MA experiments generate ROS and thus more oxidized bases such as *O*^6^-methylguanine (*O*^6^-meG) and 8-oxo-guanine (8-oxo-G) (48, 49). The *O*^6^-meG modification commonly results in G⋅C > A⋅T mutations, while the 8-oxo-G modification commonly results in G⋅C > T⋅A mutations. It remains unclear how either the origin or failed repair of ROS-induced lesions would be periodic with respect to replication timing. Conceivably, early-replicated sites on chr1 might be repaired more frequently by alternative pathways like translesion synthesis (48), and/or access to these repair complexes might be diluted with each new round of replication. This hypothesis could be tested by MA-WGS experiments under conditions that alter ROS exposure (44). For example, one recent experiment that focused on how the antibiotic norfloxacin influenced mutation rates in E. coli also tested effects of added peroxide because antibiotics may kill by ROS (50). Remarkably, this study also found periodic mutation rates that were mirrored on both replichores in the peroxide-treated lines, but no periodicity was seen in the norfloxacin-treated lines (potentially because of slower growth), indicating that mutation rate periodicity may be induced by cyclical ROS-mediated effects.

**FIG 6:**
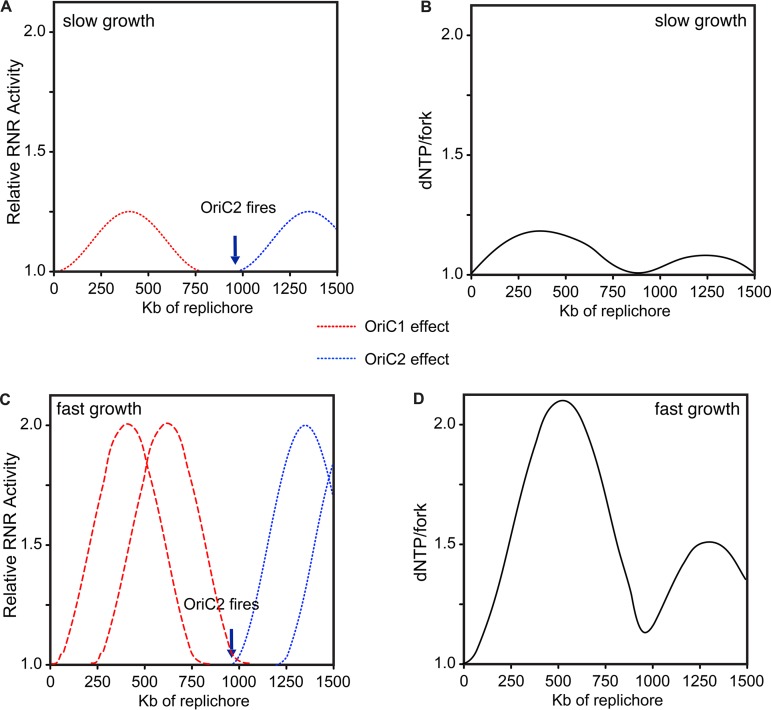
Hypothesized model of the relationship between replication timing, ribonucleotide reductase (RNR) activity, and the resulting availability of dNTPs per active replication fork. The model is fit to the V. cholerae genome with two chromosomes (chr1 and -2): one of 3.0 Mb and one of 1.1 Mb. RNR activity follows a wave that rises after the firing of the origin of chr1 and then steadily declines until additional origins fire. The chr2 origin should fire after ~950 kb of replication on each replichore of chr1 to ensure termination synchrony between chromosomes, stimulating a second wave of RNR activity. The right axis uses arbitrary relative units (dNTPs/fork) to depict how RNR activity is expected to increase dNTP pools to a maximum level (2.0) that is diluted by the number of concurrent, active forks. (A and B) Under slow growth, RNR activity rises and then falls to the baseline required to maintain synthesis. (C and D) Faster growth requires a second round of replication. Note that further rounds of overlapping replication do not significantly alter predicted dNTPs/fork, the hypothesized driver of mutation rate variability.

With these alternative explanations in mind, we suggest that the most straightforward dynamic that could produce wave-like bpsm rates is variation in levels of deoxyribonucleotides (deoxynucleoside triphosphates [dNTPs]). We describe a simple model of how dNTPs per replication fork may vary with *Vibrio* replication in Fig. 6. Synthesis of dNTPs is controlled by levels of ribonucleotide reductase (RNR), whose production is coordinated with the rate of DNA synthesis but reaches its maximum following the onset of DNA replication to meet demand (51, 52). High levels of dNTPs are mutagenic in many organisms because of increased probability of misincorporation (52–54). In slow-growing bacteria whose division rates exceed the time required for chromosome replication, dNTP availability should increase after the start of replication and transiently increase the mutation rate but then decline to a baseline (Fig. 6A and B). This predicts that slow growth should cause no mutation rate periodicity, as the results from antibiotic-limited E. coli MA lines suggest (50). However, when bacterial generation times are faster than the time required for chromosome replication, which is commonplace for fast-growing species like E. coli or *Vibrio*, new rounds of replication are initiated and proceed before the first round concludes (55). Multichromosome genomes like those of *Vibrio* species require the additional firing of *oriC2*, which generates another burst of dNTP synthesis (Fig. 6C and D). Consequently, fast-growing bacteria may experience multiple pulses of elevated RNR activity as origins fire (51), but the mutational effects of successive pulses of dNTP synthesis should be diluted across a growing number of active replication forks. We suggest this dynamic can simply generate the wave-like bpsm pattern observed in these experiments (Fig. 1 and 6) as well as those previously reported in E. coli (22). Importantly, subsequent rounds of overlapping replication of either chromosome would only marginally affect the basic periodicity because dNTPs are diluted across multiple replication forks. Furthermore, the model may explain two key features of the waves observed in our MA experiments—the greater amplitude of the first wave nearer to the origin and the lower overall variance in mutation rates in late-replicated regions, which results from dNTP bursts being diluted across more active replication forks (Fig. 3). This model may also explain why not all bacterial genomes appear to experience periodic mutation rates (23) if they grow more slowly than the time for chromosome replication. We acknowledge that this model is speculative and requires considerable additional study, although the associations between replication dynamics and RNR activity and dNTP pools and mutation rates are both well supported (53, 56–58). A related possibility is that this periodicity arises from imbalances between rNTP and dNTP pools, which have been demonstrated to be mutagenic (53, 59, 60). At a minimum, this simple model relating ribonucleotide availability to mutation rate periodicity is empirically testable by additional MA-WGS with defined mutants and altered growth conditions.

The presence of conserved patterns of bpsm rates across concurrently replicated regions of MMR^−^ lines also raises the question of whether these mutation biases influence the evolution of *Vibrio* genomes. In our previous studies of the mutation spectra from these experiments, higher rates of particular mutations were indeed found at synonymous sites among extant *Vibrio* and *Burkholderia* genomes (19, 21). If natural bpsm rates are in fact periodic in nature, we would expect genetic variation among strains to positively correlate with the bpsm rates in our defined 100-kb intervals, particularly on chr1. We calculated the average pairwise synonymous (*dS*) and nonsynonymous (*dN*) substitution rates in these intervals of V. fischeri and V. cholerae genomes (see Materials and Methods) and found a significant positive correlation for *dS* on chr1 but not on chr2 in V. fischeri (see Fig. S6 in the supplemental material). As expected from stronger selection on nonsynonymous sites, no significant correlation between *dN* and bpsm rates was found on either chromosome (Fig. S6). No significant correlations between *dS* or *dN* and bpsm rates were found on either chromosome of V. cholerae (Fig. S6). The scant correlations between evolutionary rates in coding sequences and spontaneous mutation rates may simply reflect that selection operating on both synonymous and nonsynonymous sites is quite strong in bacteria (61). Alternatively, the natural patterns of bpsm rates in V. fischeri and V. cholerae may not be consistent with those observed in MMR^−^ lines, which are strongly biased toward transition mutations. A more extensive study of the mutation spectra of wild-type genomes both experimentally and in natural isolates will determine the extent to which mutation rate periodicity shapes genome evolution.

10.1128/mBio.01371-18.7 FIG S6  Relationship between base substitution mutation rates (bpsm) with average synonymous substitution rates (left panels) and average nonsynonymous substitution rates (right panels) of genes. Average synonymous and nonsynonymous substitution rates were calculated using the average rates of all one-to-one orthologs shared between V. fischeri ES114 and MJ11 or between V. cholerae 2740-80 and HE-16 within each 100-kb interval. Synonymous and nonsynonymous substitution rates for individual genes were calculated as described by Z. Yang and R. Nielsen (Mol Biol Evol 17:32–43, 2000). In V. fischeri, only the relationship between bpsm rates and synonymous substitution rates on chromosome 1 is significant (A, chr1, *F* = 8.32, df = 28, *P* = 0.0080, *r*^2^ = 0.23, and chr2, *F* = 0.56, df = 14, *P* = 0.4681, *r*^2^ = 0.04; B, chr1, *F* = 2.14, df = 28, *P* = 0.1554, *r*^2^ = 0.07, and chr2, *F* = 0.03, df = 14, *P* = 0.8692, *r*^2^ = 0.02), and in V. cholerae, none are significant (C, chr1, *F* = 0.43, df = 28, *P* = 0.5186, *r*^2^ = 0.02, and chr2, *F* = 0.49, df = 10, *P* = 0.5010, *r*^2^ = 0.05; D, chr1, *F* = 0.02, df = 28, *P* = 0.8897, *r*^2^ = 0.01 × 10^−1^, and chr2, *F* = 0.01, df = 10, *P* = 0.9218, *r*^2^ = 0.01 × 10^−1^). Download FIG S6, PDF file, 0.5 MB. Copyright © 2018 Dillon et al. 2018 Dillon et al. This content is distributed under the terms of the Creative Commons Attribution 4.0 International license.

In summary, we have shown that bpsm rates in MMR-deficient lineages of V. cholerae *and* V. fischeri are nonuniformly distributed on chr1 and vary in a mirrored wave-like pattern that extends bidirectionally from the origin of replication. In contrast, late-replicated regions of chr1 and the entirety of chr2 experience more constant bpsm rates. These observations suggest that concurrently replicated regions of bacterial genomes experience similar bpsm rates prior to MMR, which could be governed by a number of temporally regulated cellular processes, including ROS, variation in dNTP pools, and the availability of replication machinery with secondary rounds of replication. We encourage research to disentangle effects of these cellular processes on bpsm rates (see reference 62, for example), as well as the signatures of these processes in natural populations, which will deepen our understanding of how mutation rates vary within genomes. Recalling that the relative distance of genes from the origin of replication is highly conserved across broad phylogenetic distances for a variety of functional reasons (6), it is quite possible that some genes are exposed to an elevated mutational load, while others are more shielded. In light of the growing effort toward evolutionary forecasting in microbial genomes (63), the need to determine whether the probability of new mutations substantively differs between genome regions is all the more pressing.

## MATERIALS AND METHODS

### Bacterial strains and culture conditions.

MMR-deficient ancestors were generated by replacing the *mutS* gene in V. fischeri ES114 and V. cholerae 2740-80 with an erythromycin resistance cassette, as described previously (64–67). Complete genome sequences of these ancestors are publicly available (68, 69) or were generated by us for this project (70). Replication origins were determined using Ori-Finder (19, 71, 72).

MA experiments with both the V. fischeri MMR^−^ and wild-type strains were conducted on tryptic soy agar (TSA) plates plus NaCl (30 g/liter tryptic soy broth powder, 20 g/liter NaCl, 15 g/liter agar) incubated at 28°C. MA experiments with the V. cholerae MMR^−^, V. cholerae wild type, and B. cenocepacia wild type were conducted on TSA (30 g/liter tryptic soy broth powder, 15 g/liter agar) and incubated at 37°C. MA experiments with MMR^−^ lines involved 48 independent lineages founded from single colonies of V. fischeri *mutS* or V. cholerae *mutS* cells and were propagated daily for 43 days. MA experiments with WT lines involved 75 lineages founded from single colonies of V. fischeri, V. cholerae, or B. cenocepacia and were propagated daily for 217 days (19, 21).

### Base substitution mutation rate analysis at different genome intervals.

Genomes were divided into intervals of 10, 25, 50, 100, 250, and 500 kb, and bpsms were categorized by interval and location. On chr1, these intervals start at *oriCI* and extend bidirectionally to the replication terminus to mimic the progression of the two replication forks. Rates of bpsm were analyzed on secondary chromosomes similarly, but intervals were measured relative to the initiation of replication of *oriCI* rather than to *oriCII* (Fig. S1). This enables direct comparisons between concurrently replicated intervals on chr1 and chr2 based on established models of secondary chromosome replication timing in V. cholerae (28, 32, 34). Matched intervals of the same length were defined on each chromosome. (Note that chromosomes are not perfectly divisible by interval lengths, so some intervals are shorter.) bpsm rates in each interval were calculated as the number of mutations observed in each interval divided by the product of the total number of sites analyzed in that interval across all lines and the total number of generations of mutation accumulation, so rates in shorter intervals could be directly compared to the full-length intervals. For independent analyses of A⋅T > G⋅C and G⋅C > A⋅T mutations, bpsm rates were calculated as the number of mutations observed in each interval divided by the product of the total number of sites in that interval that could lead to the bpsm being analyzed (A+T sites for A⋅T > G⋅C and G+C sites for G⋅C > A⋅T) and the total number of generations of mutation accumulation.

### Wavelet transformations.

We used the R package WaveletComp to evaluate properties of the wave-like patterns in bpsm rates in V. fischeri and V. cholerae and to test whether waves on opposing replichores were synchronous (35). The periodicity of bpsm rates on each chromosome of the V. fischeri MMR^−^ and V. cholerae MMR^−^ lineages at an interval length of 100 kb was analyzed, treating each chromosome as a univariate series starting at the origin of replication and extending clockwise around the chromosome. WaveletComp uses the Morlet wavelet to transform the series of mutation rates then tests the null hypothesis of no periodicity for all combinations of intervals and periods (35). We performed this analysis using the “white.noise” method, with no smoothing, and a period range of 0.2 Mb to the entire length of the respective chromosomes. Default settings were used for all other parameters.

To test whether opposing replichores on chr1 here synchronous, we used a cross-wavelet transformation (35) to test the null hypothesis that no joint periodicity (synchronicity) exists among the two series as they traverse the primary chromosome in opposite directions. We used default settings but turned off smoothing and specified a period range of 0.2 Mb to the entire length of chr1 in both V. fischeri and V. cholerae.

### Sequencing and mutation identification.

Methods for genome sequencing, mutation identification, and evolutionary rate analyses are described in Text S1 in the supplemental material.

### Data availability.

The accession numbers for all of the whole-genome sequencing data produced by this study are PRJNA256340 for V. fischeri, PRJNA256339 for V. cholerae, and PRJNA326274 for B. cenocepacia.
