# Supplementary material for: Periodic Variation of Mutation Rates in Bacterial Genomes Associated with Replication Timing
Source: mBio. 2018 Aug 21;9(4):e01371-18. doi: 10.1128/mBio.01371-18 (PMC6106087; doi:10.1128/mBio.01371-18)
Supplement: FIG S1 [file mbo004184027sf1.pdf]

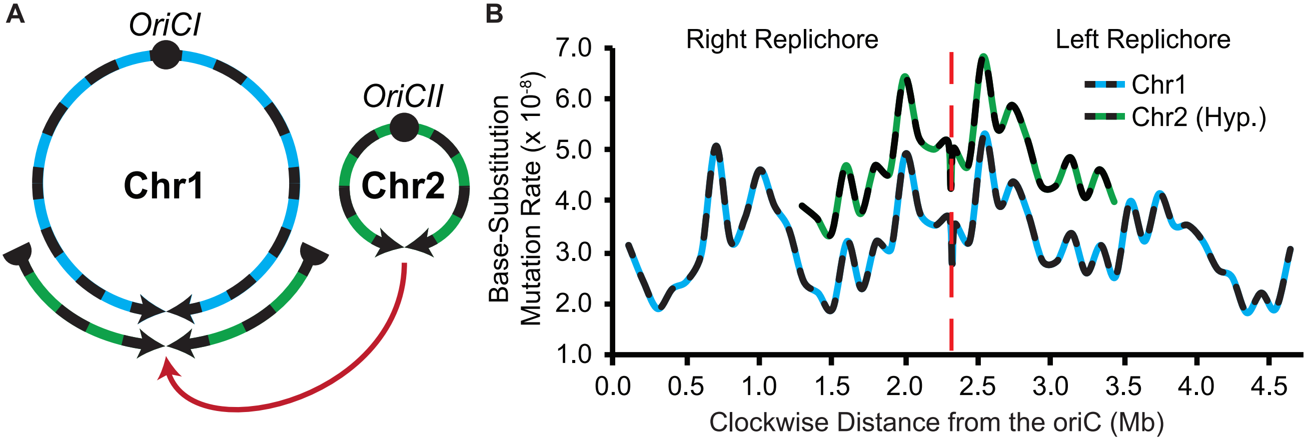


**Figure S1**. Design of the interval analysis used in this study to enable direct comparisons of base-substitution mutation (bpsm) rates of concurrently replicated regions on chromosome 1 (chr1) and chromosome 2 (chr2). A) For all multi-chromosome species analyzed in this study, secondary chromosomes are split at their origin of replication (*oriCII*), and mapped directly to concurrently replicated intervals in late replicating regions of chr1. All intervals on both chromosomes are thus relative to the initiation of replication of *oriCI*, and the boundaries of the intervals are consistent with their replication timing. B) Patterns of bpsm rates on the single chromosome of *Escherichia coli* MG1655 rph+ ΔmutL, derived from (19), show a wave-like mirrored pattern of bpsm rates on the two opposing replichores. If replication timing governs this pattern, a hypothetical secondary chromosome would be expected to mirror patterns of bpsm rates of late replicated regions on the primary chromosome.
