# Supplementary material for: Periodic Variation of Mutation Rates in Bacterial Genomes Associated with Replication Timing
Source: mBio. 2018 Aug 21;9(4):e01371-18. doi: 10.1128/mBio.01371-18 (PMC6106087; doi:10.1128/mBio.01371-18)
Supplement: FIG S2 [file mbo004184027sf2.pdf]

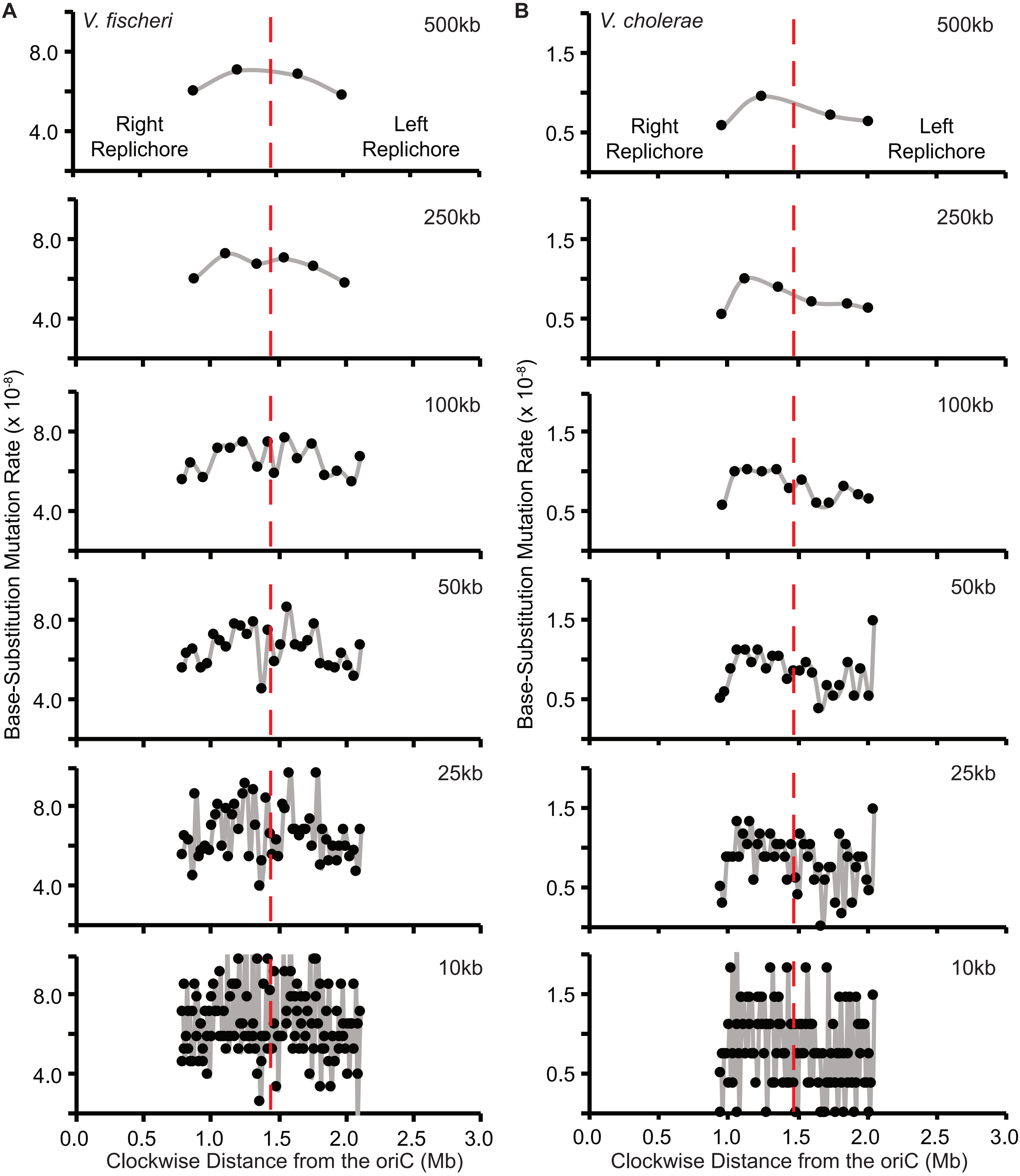

Figure S2. Patterns of base-substitution mutation (bpsm) rates at various size intervals extending clockwise from the origin of replication (*oriCII*), in MMR-deficient mutation accumulation lineages of *Vibrio fischeri* (A) and *Vibrio cholerae* (B) on chromosome 2. All interval breakpoints are plotted relative to the initiation of replication of *oriCI* so that the boundaries of the intervals are at identical locations.
