# Supplementary material for: Periodic Variation of Mutation Rates in Bacterial Genomes Associated with Replication Timing
Source: mBio. 2018 Aug 21;9(4):e01371-18. doi: 10.1128/mBio.01371-18 (PMC6106087; doi:10.1128/mBio.01371-18)
Supplement: FIG S3 [file mbo004184027sf3.pdf]

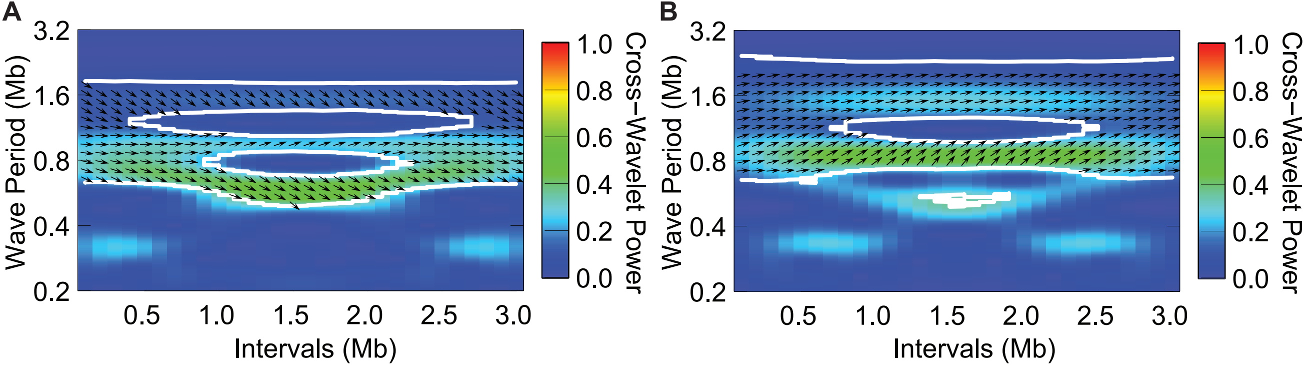

Figure S3. Cross-wavelet power spectrum plots comparing the patterns of base-substitution mutation (bpsm) rates in 100 Kb intervals extending clockwise from the *oriCI* region to those extending counterclockwise from the *oriCI* region in MMR-deficient mutation accumulation lineages of *Vibrio fischeri* (A) and *Vibrio cholerae* (B). Plots were generated using the WaveletComp package for Computational Wavelet Analysis in R, using an interval color key, 100 simulations, and significant synchronicity cutoffs of $p < 0.1$ for contour (white lines) and $p < 0.05$ for arrows.
