# Supplementary material for: Periodic Variation of Mutation Rates in Bacterial Genomes Associated with Replication Timing
Source: mBio. 2018 Aug 21;9(4):e01371-18. doi: 10.1128/mBio.01371-18 (PMC6106087; doi:10.1128/mBio.01371-18)
Supplement: FIG S4 [file mbo004184027sf4.pdf]

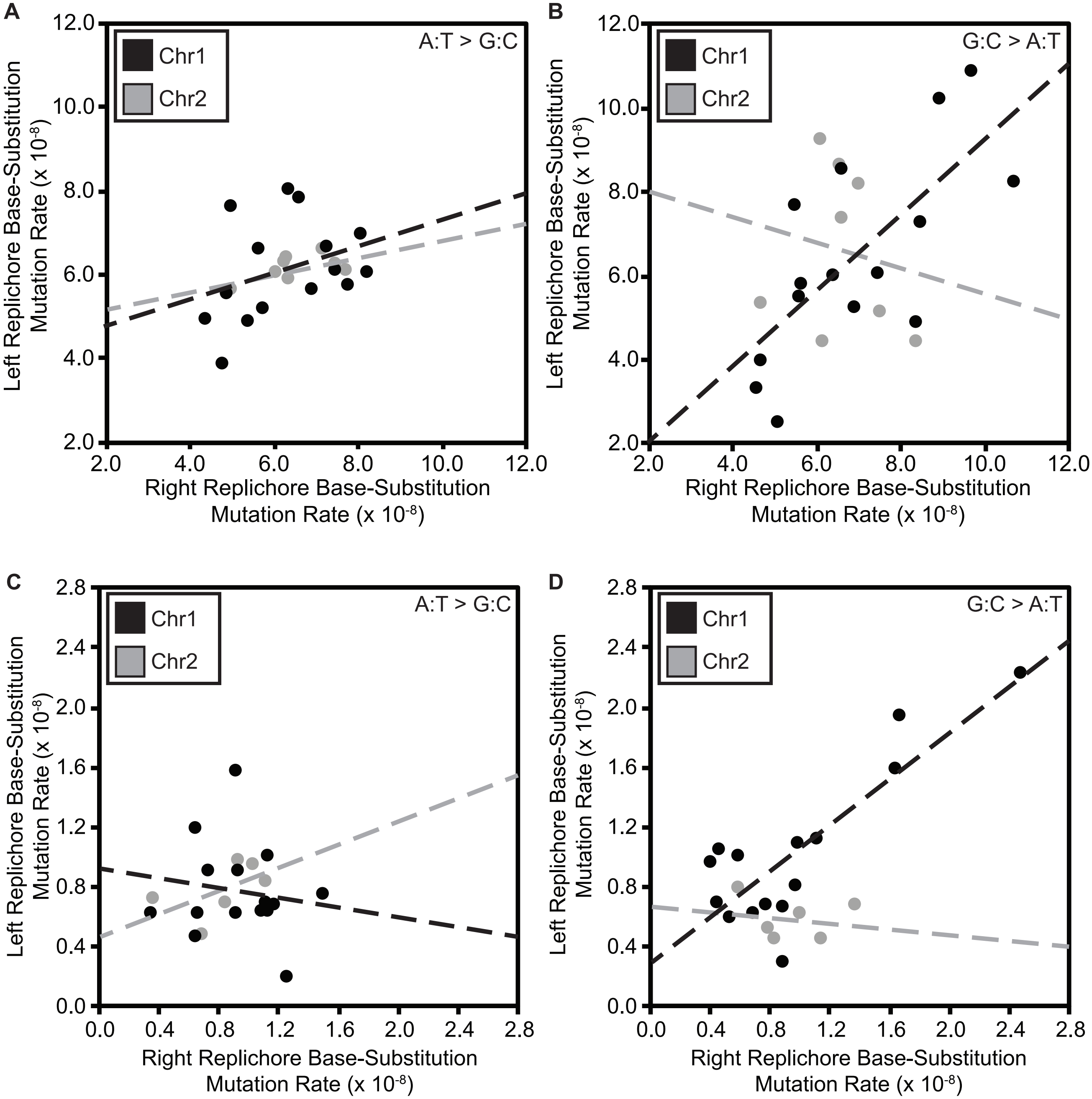

Figure S4: Relationship between base-substitution mutation (bpsm) rates in 100 Kb intervals on the right replichore with concurrently replicated 100 Kb intervals on the left replichore for A:T>G:C (A) and G:C>A:T (B) bpsms in MMR-deficient Vibrio fischeri and C) A:T>G:C and D) G:C>A:T bpsms in MMR-deficient Vibrio cholerae. Only the relationship between G:C>A:T bpsm rates of concurrently replicated regions on chr1 is significantly positive (Vf: A:T>G:C: Chr1 - F = 1.77, df = 13, p = 0.2067, r2 = 0.12, Chr2 - F = 3.26, df = 6, p = 0.1209, r2 = 0.35; G:C>A:T: Chr1 - F = 13.32, df = 13, p = 0.0029, r2 = 0.51, Chr2 - F = 0.17, df = 6, p = 0.6947, r2 = 0.03; Vc: A:T>G:C: Chr1 - F = 0.24, df = 13, p = 0.6313, r2 = 0.02, Chr2 - F = 1.74, df = 4, p = 0.2574, r2 = 0.30; G:C>A:T: Chr1 - F = 28.99, df = 13, p = 0.0001, r2 = 0.6904, Chr2 - F = 0.15, df = 4, p = 0.7209, r2 = 0.04).
