# Supplementary material for: Periodic Variation of Mutation Rates in Bacterial Genomes Associated with Replication Timing
Source: mBio. 2018 Aug 21;9(4):e01371-18. doi: 10.1128/mBio.01371-18 (PMC6106087; doi:10.1128/mBio.01371-18)
Supplement: FIG S5 [file mbo004184027sf5.pdf]

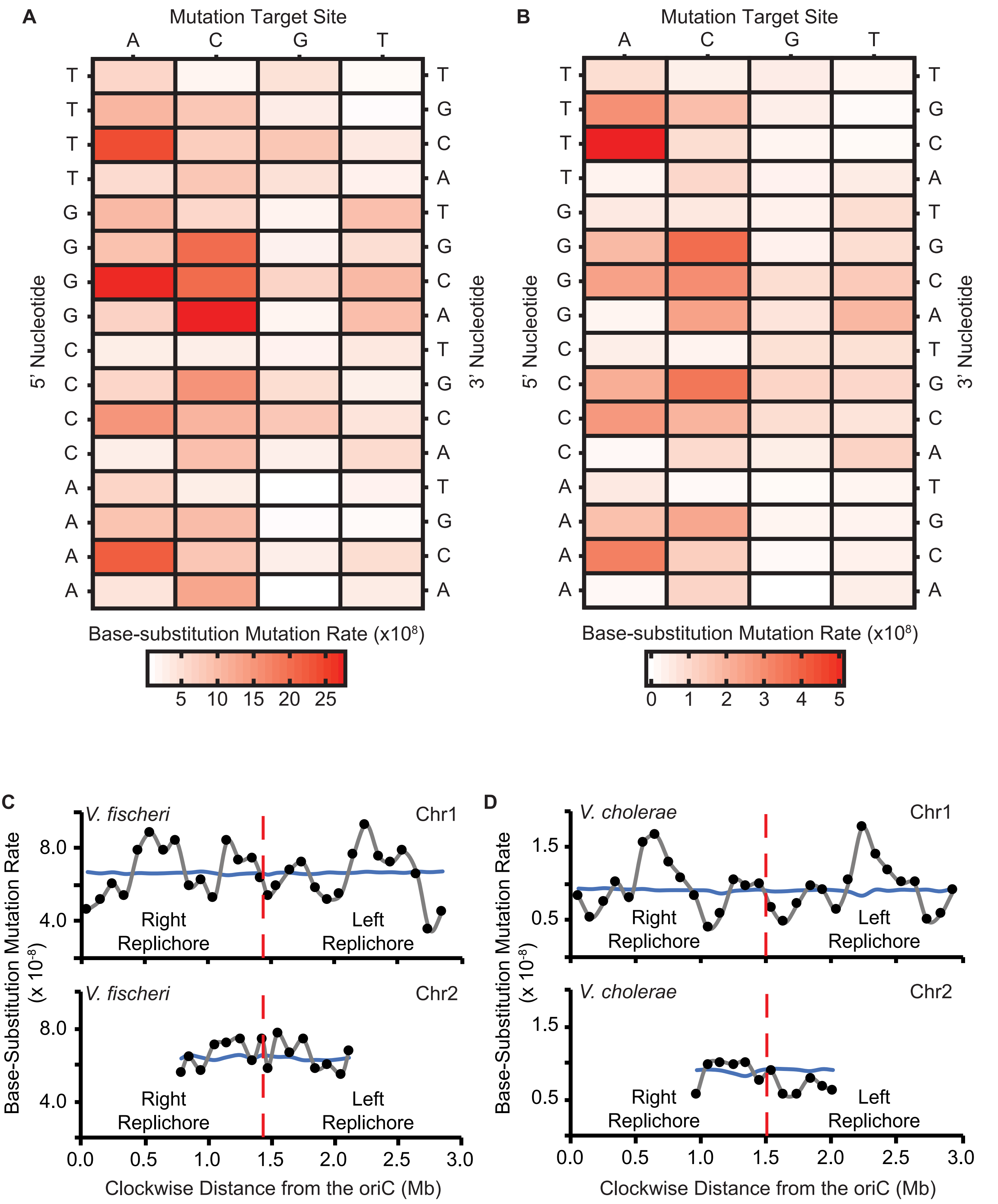


**Figure S5**. Effects of nucleotide context (trimer content) on bpsm rates. A) Heatmap of the context dependent base-substitution mutation (bpsm) rates for the 64 possible trimer combinations based on their lagging strand orientation in MMR-deficient mutation accumulation lineages of *V. fischeri* (A) and *V. cholerae* (B). B) Patterns of base-substitution mutation (bpsm) rates in 100 Kb intervals extending clockwise from the origin of replication (oriCI) in MMR-deficient mutation accumulation lineages of *V. fischeri* (C) and *V. cholerae* (D). Observed patterns of bpsm rates (gray lines) on chromosome 1 (Chr1) and chromosome 2 (Chr2) are compared to the expected patterns of bpsm rates (blue lines) based on the trimer content of the interval. Bpsm rates differ significantly from expectations based on trimer content: Chi-square test; Vf-mut: Chr1 - $\chi 2 = 137.24$, df = 29, $p < 0.0001$ , Chr2 - $\chi 2 = 20.04$, df = 15, $p = 0.1703$; Vc-mut: Chr1 - $\chi 2 = 107.55$, df = 29, $p < 0.0001$, Chr2 - $\chi 2 = 14.87$, df = 1, $p = 0.1887$)
