# Supplementary material for: Periodic Variation of Mutation Rates in Bacterial Genomes Associated with Replication Timing
Source: mBio. 2018 Aug 21;9(4):e01371-18. doi: 10.1128/mBio.01371-18 (PMC6106087; doi:10.1128/mBio.01371-18)
Supplement: TEXT S1 [file mbo004184027s1.docx]

**Supplemental Methods**

**MA-WGS Process.** To estimate daily generation times, ten representative colonies following 24 hours of growth were placed in 2 ml of phosphate buffer saline, serially diluted, and spread plated on the appropriate media (see above) to calculate the number of viable cells in each colony. The number of generations elapsed over 24 hours of growth was then calculated, and the average number of generations across the ten representative colonies was used as the experiment-wide daily generations for each lineage at that time-point. The total generations elapsed between each measurement was calculated as the product of the average daily generations and the number of days before the next measurement, and the total of number of generations elapsed during the entire MA experiment, per lineage, was calculated as the sum of these totals. At the conclusion of each MA experiment, all lineages were stored at -80° in 8% DMSO and later revived for WGS.

Genomic DNA was extracted from 1 ml of overnight culture (TSBN at 28° for *V. fischeri*; TSB at 37° for *V. cholerae* and *B. cenocepacia*) in 50 representative lineages from the three wild-type experiments and 48 lineages from the two mutator experiments using the Wizard Genomic DNA Purification Kit (Promega). All libraries were prepared using a modified Illumina Nextera protocol designed for inexpensive library preparation of microbial genomes (1). Sequencing of the *Vf*-mut, *Vc*-mut, and *Bc*-wt lineages was performed using the 151-bp paired-end Illumina HISeq platform at the University of New Hampshire Hubbard Center for Genomic Studies, while sequencing for the *Vf*-wt and *Vc*-wt lineages was performed using the 101-bp paired-end Illumina HiSeq platform at the Beijing Genome Institute (BGI). In sum, we analyzed 19 *Vf*-mut lineages, 22 *Vc*-mut lineages, 48 *Vf*-wt lineages, 49 *Vc*-wt lineages, and 47 *Bc*-wt lineages, as fastQC revealed that the depth of coverage for the other sequenced lineages was insufficient for accurate detection of polymorphism (2). The reads from each of these lineages were mapped to their respective reference genomes with the Burrows-Wheeler Aligner (BWA) (3) and Novoalign (www.novocraft.com). The average depth of coverage was 124x for *Vf*-mut, 92x for *Vc*-mut, 100x for *Vf*-wt, 96x for *Vc*-wt, and 50x for *Bc*-wt.

**Base-substitution mutation identification.** For each MA experiment, bpsms were identified as described previously (4, 5). Briefly, we used SAMtools to convert the SAM alignment files from each lineage to mpileup format (6), then in-house perl scripts to produce the forward and reverse read alignments for each position in each line. A three-step process was then used to detect putative bpsms. First, pooled reads across all lines were used to generate an ancestral consensus base at each site in the reference genome. This allows us to correct for any differences that may exist between the reference genomes and the ancestral colony of each our MA experiments. Second, a lineage specific consensus base was generated at each site in the reference genome for each individual MA lineage using only the reads from that line. Here, a lineage specific consensus base was only called if the site was covered by at least two forward and two reverse reads and at least 80% of the reads identified the same base. Otherwise, the site was not analyzed. Third, each lineage specific consensus base that was called was compared to the overall ancestral consensus of the MA experiment and a putative bpsm was identified if they differed. This analysis was carried out independently with the alignments generated by BWA and Novoalign, and putative bpsms were considered genuine only if both pipelines independently identified the bpsm and they were only identified in a single lineage. All genuine bpsms analyzed in this study are summarized in Supplementary Dataset S1, which shows that nearly all bpsms were identified with high-confidence and were not clustered at the lower limits of detection.

**Evolutionary rate analysis at different interval lengths.** As we did with bpsm rates, we estimated extant sequence variation in the *V. fischeri* ES114 and *V. cholerae* 2740-80 genomes using the average synonymous (dS) and non-synonymous (dN) substitution rates of genes in each 100 Kb interval extending bi-directionally from the two origins of replication. Specifically, for *V. fischeri*, we measured dS and dN of all one-to-one orthologs shared between *V. fischeri* ES114 and *V. fischeri* MJ11, which have 83.66% symmetric identity. For *V. cholerae*, we measured dS and dN of all one-to-one orthologs shared between *V. cholerae* 2740-80 and *V. cholerae* HE-16, which have 84.54% symmetric identity. The genomes of *V. fischeri* MJ11 and *V. cholerae* HE-16 were chosen because they were sufficiently diverse relative to *V. fischeri* ES114 and *V. cholerae* 2740-80 to produce reliable evolutionary rate measurements, while also maintaining the synteny of the majority of the one-to-one orthologs.

To calculate evolutionary rates for all one-to-one orthologs, we first identified all orthologs using orthoMCL with default settings (7). We then aligned all one-to-one orthologs using the Needleman-Wunsch algorithm with a gap open penalty of 10 and a gap extend penalty of 0.5 (8), and converted these amino-acid alignments to nucleotide alignments using RevTrans (9). Pairwise evolutionary rates were then computed in paml (10), using the Yang and Nielson method for pairwise substitution rate estimates (11). Lastly, any one-to-one orthologs with dS or dN values exceeding 2.0 were discarded because saturation can render these values unreliable. In sum, we used evolutionary rate estimates of 3,259 one-to-one orthologs shared by *V. fischeri* ES114 and *V. fischeri* MJ11, and 2,849 one-to-one orthologs shared by *V. cholerae* 2740-80 and *V. cholerae* HE-16. Estimates of dS and dN in 100 Kb intervals equivalent to those described above for bpsm rates were calculated as the average evolutionary rates of all one-to-one orthologs in each interval. In the rare cases where orthologs overlapped across two intervals, the ortholog was considered to be part of the interval in which its start codon resides.

**Power analysis of these studies to detect significant regional variability in mutation rates:**

Effect size (*w*) was estimated for the two mutator experiments as:

$$w= \sqrt{\sum_{i=1}^{m} \frac{{(e_{i}- o_{i})}^{2}}{e_{i}}}$$

, where *e_i_* is the expected proportion of mutations in each 100 Kb interval and *o_i_* is the observed proportion of mutations in each 100 Kb interval. We estimate a *w* of 0.2138 for chr1 in the *Vf*-mut experiments and 0.3631 for chr1 in the *Vc*-mut experiments, where mutation rates were non-uniformly distributed.

**Other Statistical analyses.** All statistical analyses were performed in R Studio Version 1.1.383 using the Stats analysis package (12).

**Supplementary References**

1. Baym M, Kryazhimskiy S, Lieberman TD, Chung H, Desai MM, Kishony R. 2015. Inexpensive multiplexed library preparation for megabase-sized genomes. Plos One 10:e0128036.

2. Andrews S. 2010. FastQC: A quality control tool for high throughput sequence data.

3. Li H, Durbin R. 2009. Fast and accurate short read alignment with Burrows-Wheeler transform. Bioinformatics 25:1754–1760.

4. Dillon MM, Sung W, Lynch M, Cooper VS. 2015. The rate and molecular spectrum of spontaneous mutations in the GC-rich multichromosome genome of *Burkholderia cenocepacia*. Genetics 200:935–946.

5. Dillon MM, Sung W, Lynch M, S. C V. 2017. Genome-wide biases in the rate and molecular spectrum of spontaneous mutations in *Vibrio cholerae* and *Vibrio fischeri*. Mol Biol Evol 34:93–109.

6. Li H, Handsaker B, Wysoker A, Fennell T, Ruan J, Homer N, Marth G, Abecasis G, Durbin R. 2009. The sequence alignment/map format and SAMtools. Bioinformatics 25:2078–2079.

7. Li L, Stoeckert CJJ, Roos DS. 2003. OrthoMCL: Identification of ortholog groups for eukaryotic genomes. Genome Res 13:2178–2189.

8. Rice P, Longden I, Bleasby A. 2000. EMBOSS: The European Molecular Biology Open Software Suite. Trends Genet 16:276–277.

9. Wernersson R, Pedersen AG. 2003. RevTrans: Multiple alignment of coding DNA from aligned amino acid sequences. Nucleic Acids Res 31:3537–3539.

10. Yang ZH. 2007. PAML 4: Phylogenetic analysis by maximum likelihood. Mol Biol Evol 24:1586–1591.

11. Yang Z, Nielsen R. 2000. Estimating synonymous and nonsynonymous substitution rates under realistic evolutionary models. Mol Biol Evol 17:32–43.

12. R Development Core Team. 2011. R: A language and environment for statistical computing. R Foundation for Statistical Computing, Vienna, Austria.
