# Supplementary material for: Periodic Variation of Mutation Rates in Bacterial Genomes Associated with Replication Timing
Source: mBio. 2018 Aug 21;9(4):e01371-18. doi: 10.1128/mBio.01371-18 (PMC6106087; doi:10.1128/mBio.01371-18)
Supplement: FIG S6 [file mbo004184027sf6.pdf]

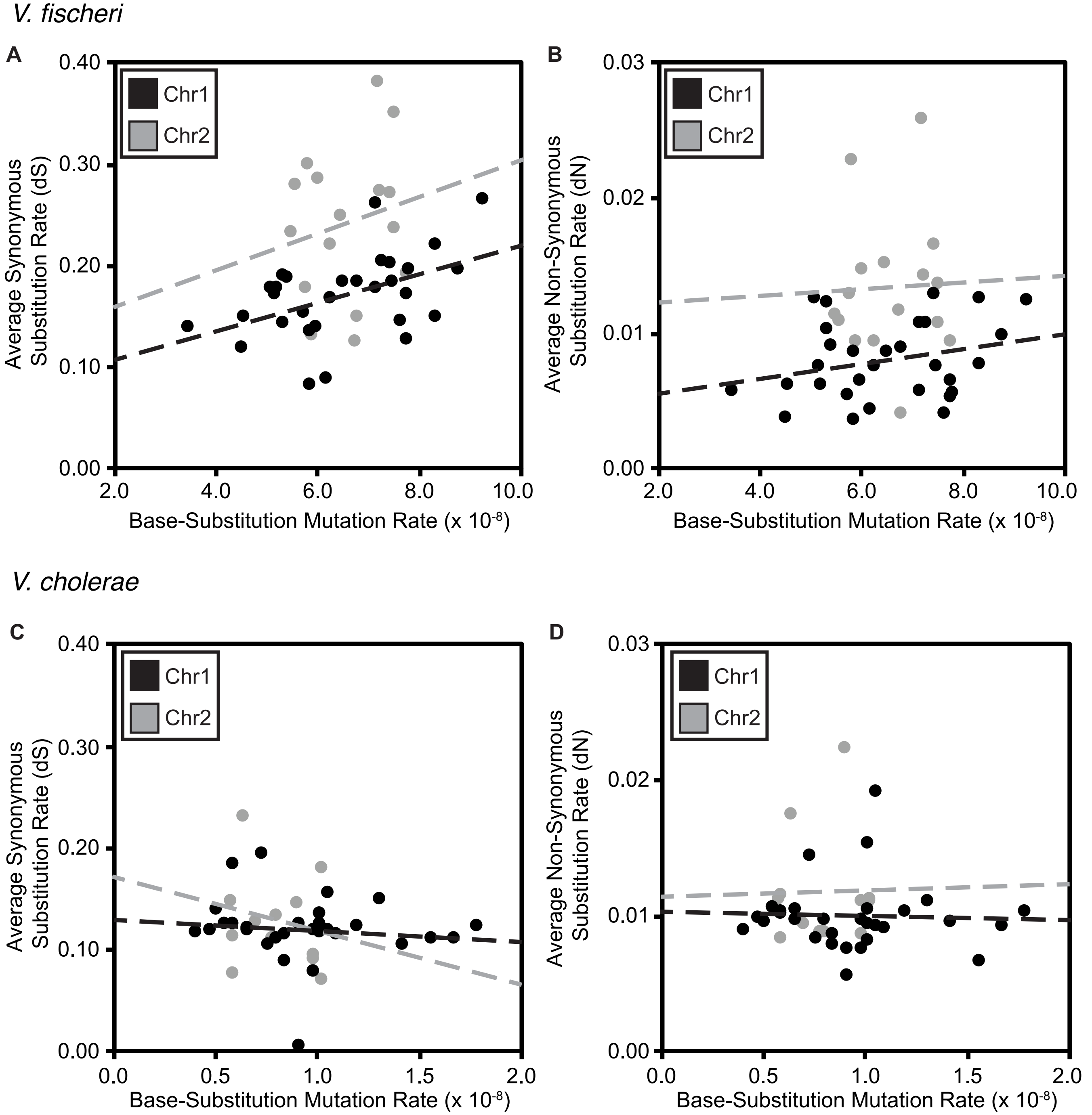

Figure S6. Relationship between base-substitution mutation rates (bpsm) with average synonymous substitution rates (left panels) and average non-synonymous substitution rates (right panels) of genes. *V. fischeri,* top row, *V. cholerae,* bottom row, Average synonymous and non-synonymous substitution rates were calculated using the average rates of all one-to-one orthologs shared between *V. fischeri* ES114 and *V. fischeri* MJ11, or between *V. cholerae* 2740-80 and *V. cholerae* HE-16 within each 100kb interval. Synonymous and non-synonymous substitution rates for individual genes were calculated as described in (Yang and Nielsen 2000). In V. fischeri, only the relationship between bpsm rates and synonymous substitution rates on chromosome 1 is significant (A: Chr1 - F = 8.32, df = 28, p = 0.0080, r2 = 0.23, Chr2 - F = 0.56, df = 14, p = 0.4681, r2 = 0.04; B: Chr1 - F = 2.14, df = 28, p = 0.1554, r2 = 0.07, Chr2 - F = 0.03, df = 14, p = 0.8692, r2 = 0.02), and in V. cholerae, none are significant (C: Chr1 - F = 0.43, df = 28, p = 0.5186, r2 = 0.02, Chr2 - F = 0.49, df = 10, p = 0.5010, r2 = 0.05; D: Chr1 - F = 0.02, df = 28, p = 0.8897, r2 = 0.01 10-1, Chr2 - F = 0.01, df = 10, p = 0.9218, r2 = 0.01 10-1).
